# Extending digital PCR analysis by modelling quantification cycle data

**DOI:** 10.1186/s12859-016-1275-3

**Published:** 2016-10-12

**Authors:** Philip J. Wilson, Stephen L. R. Ellison

**Affiliations:** LGC, Queens Road, Teddington, Middlesex TW11 0LY UK

**Keywords:** Bayesian, MCMC, Conway-Maxwell-Poisson distribution, CMP distribution, Amplification efficiency, ssDNA

## Abstract

**Background:**

Digital PCR (dPCR) is a technique for estimating the concentration of a target nucleic acid by loading a sample into a large number of partitions, amplifying the target and using a fluorescent marker to identify which partitions contain the target. The standard analysis uses only the proportion of partitions containing target to estimate the concentration and depends on the assumption that the initial distribution of molecules in partitions is Poisson. In this paper we describe a way to extend such analysis using the quantification cycle (C_q_) data that may also be available, but rather than assuming the Poisson distribution the more general Conway-Maxwell-Poisson distribution is used instead.

**Results:**

A software package for the open source language R has been created for performing the analysis. This was used to validate the method by analysing C_q_ data from dPCR experiments involving 3 types of DNA (attenuated, virulent and plasmid) at 3 concentrations. Results indicate some deviation from the Poisson distribution, which is strongest for the virulent DNA sample. Theoretical calculations indicate that the deviation from the Poisson distribution results in a bias of around 5 % for the analysed data if the standard analysis is used, but that it could be larger for higher concentrations. Compared to the estimates of subsequent efficiency, the estimates of 1st cycle efficiency are much lower for the virulent DNA, moderately lower for the attenuated DNA and close for the plasmid DNA. Further method validation using simulated data gave results closer to the true values and with lower standard deviations than the standard method, for concentrations up to approximately 2.5 copies/partition.

**Conclusions:**

The C_q_-based method is effective at estimating DNA concentration and is not seriously affected by data issues such as outliers and moderately non-linear trends. The data analysis suggests that the Poisson assumption of the standard approach does lead to a bias that is fairly small, though more research is needed. Estimates of the 1st cycle efficiency being lower than estimates of the subsequent efficiency may indicate samples that are mixtures of single-stranded and double-stranded DNA. The model can reduce or eliminate the resulting bias.

**Electronic supplementary material:**

The online version of this article (doi:10.1186/s12859-016-1275-3) contains supplementary material, which is available to authorized users.

## Background

Digital Polymerase Chain Reaction (dPCR) is a technique first published in [1] that is used to quantify deoxyribonucleic acid (DNA) and other nucleic acids such as ribonucleic acid (RNA) for a variety of applications such as absolute quantification [2], copy number variation [3] and rare mutation detection [4]. It is now being used as a reference method to assign the copy number concentration of reference materials [5]. Samples are loaded onto a chip in a large number of separate partitions and then a series of cycles of the Polymerase Chain Reaction (PCR) are used to amplify the nucleic acid in the partitions. Fluorescent markers are used to detect which partitions contain nucleic acid.

The most basic data produced by this process are the counts of positive and negative reactions. These count data are sufficient, under the standard assumption [1] that the molecules in the partitions are initially independently distributed following a Poisson distribution [6], to calculate an estimate of the concentration of the target nucleic acid. The estimate for the mean molecules per partition based on the Poisson assumption is

1 $$ \tilde{\mu}=- \log \left(\frac{n_0}{n}\right) $$

where *n*_0_ is the number of negative partitions out of a total of *n* and log refers to the natural logarithm. This estimate follows the classical statistics (also called frequentist statistics) method of maximum likelihood. If the Poisson distribution assumption is invalid then the estimate is likely to be biased.

In some dPCR instruments, the fluorescence is measured after each PCR cycle in what is known as real-time dPCR. The data are processed to produce the amplification curve for each partition, which for positive partitions includes a phase of exponential growth and, eventually, a plateau with no further growth. This provides a measure of fluorescence as a proxy for the amount of the target at each cycle, and is used to calculate the quantification cycle (C_q_) for each positive partition. This is defined as the cycle at which fluorescence reaches a fixed threshold [7], with cycle treated as a continuous variable. The threshold is chosen so that it is crossed during the phase when fluorescence is growing exponentially. A common method is to fit a curve to the data and calculate the point at which it crosses the threshold. Such data have the potential to provide more information than the counts do, particularly about the value and uncertainties of the relevant concentration.

One approach to analysing C_q_ data is the retroflex method described in [8], where a continuous extension of the Poisson distribution is used to approximate the distribution of the data. In this paper we describe and illustrate a method of analysing C_q_ data from dPCR experiments that is appropriate for concentrations up to approximately 2.5 copies/partition, and that allows for possible departures from the Poisson distribution.

## Methods

### Model

The standard method requires the assumption of a single parameter distribution such as the Poisson distribution because the simple count data only provide information about whether the numbers of initial molecules in partitions are either zero or at least one. The justification for the Poisson distribution comes from the Poisson limit theorem which in part depends on the independence of the positions of the DNA molecules within the fluid. If there are significant dependencies, for example due to molecules sticking together or repelling each other, then there may be some deviation from the Poisson distribution. This may depend on factors such as the length of the DNA strands and the partition size.

The Poisson distribution has probability mass function

2 $$ P\left(X=x;\mu \right)={e}^{-\mu}\frac{\mu^x}{x!},\ x=0,\ 1,\dots,\ \mu >0. $$

A less restrictive distribution is the Conway-Maxwell-Poisson (CMP) distribution [9], which has probability mass function

3 $$ P\left(X=x;\lambda, \nu \right)=\frac{1}{Z\left(\lambda, \nu \right)}\frac{\lambda^x}{{\left(x!\right)}^{\nu }},\ x=0,\ 1,\dots; \lambda >0,\ \nu \ge 0, $$

where *Z*(*λ*, *ν*) is the normalising constant. For *ν* = 1 it is equivalent to the Poisson distribution, and the variance equals the mean. For *ν* < 1 the variance is greater than the mean and for *ν* > 1 the variance is less.

**Fig. 1:**
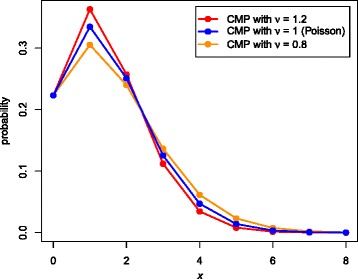
Comparison of Poisson and CMP distributions with *P*(*X* = 0) the same for each. Probabilities are given by (1) for the Poisson distribution and (2) for the CMP distribution, with *λ* chosen so that *P*(*X* = 0|*λ*, *ν*) = *e* ^− 1.5^

Figure 1 provides a comparison between the Poisson and CMP distributions, where *P*(*X* = 0) is the same for each. The means are 1.40 (CMP with *v* = 0.8), 1.50 (Poisson) and 1.62 (CMP with *v* = 1.2).

Our model of C_q_ data first requires a model of the growth of the number of molecules over the PCR amplification cycles. If the number of molecules at cycle *c* is given by *N*(*c*), then for *c* > 0

4 $$ N(c)=N\left(c - 1\right)+\mathrm{Binom}\left(N\left(c-1\right),{E}_c\right) $$

where Binom(*n*, *p*) represents a binomial random variable with *n* trials and probability *p* of success and *E*_*c*_ is the efficiency at cycle *c*. This is because each of the *N*(*c* – 1) molecules from the previous cycle is duplicated with probability *E*_*c*_.

In the model the efficiency for the first cycle is *E*_1_ but for subsequent cycles is *E*. Equation (4) can be used with Eq. (3) as the initial distribution of molecules to calculate the distribution after a chosen modest number of cycles. The distribution after further growth is modelled as following a normal distribution. The fact that Eq. (4) represents a Galton-Watson branching process [10] is used to derive the mean and variance. The introduction of the parameter *A*, defined as the relative fluorescence per molecule, leads to a distribution for relative fluorescence. This can then be used to derive an approximation for the distribution of C_q_ data for a given threshold value *h*.

The default C_q_ values provided by the data analysed later show clear trends. Additional analysis suggested that the trends could be removed through normalising the amplification curves and then calculating the C_q_ values (see Additional file 1). This approach could not be properly tested as the amplification curves generally appeared to be a few cycles short of reaching the plateau stage. It is not obvious what causes the differing plateaus, though one potential factor is varying temperature across the panels. The trends appear approximately linear in many cases, and so a linear trend is included in the model.

Censoring may be required for outliers, as they can represent some technical deviation from the model. The exclusion of such values that are inconsistent with the model should improve the performance of the analysis and the accuracy of the results. High outliers may be caused by a problem in amplifying the molecule. Our model censors high outliers, treating them as partitions with one molecule, rather than using the C_q_ values. The model could be similarly extended to deal with low outliers by treating them as counts of partitions with more than one molecule, though this was not done for the present study. As discussed later, low outliers do lead to spurious results for one of the analysed data sets.

**Fig. 2:**
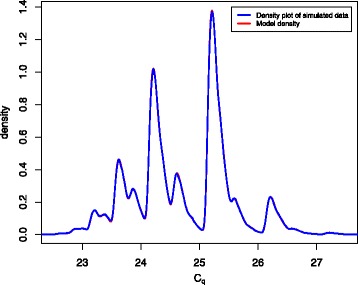
Density plot of simulated data superimposed on model density. Simulation was performed using the rcq function from the R package edpcr for *N* = 10^6^ partitions with *E* = .95, *E*_1_ = .85, *ν* = 1.2, *μ* = 1.5 and *c* = 25.5 (a location parameter used to calculate *A*). This used Eq. 3 to select *N*(0) and repeated applications of Eq. 4 to simulate subsequent growth. C_q_ values were calculated based on exponential growth between the cycles immediately before and after the threshold was crossed. The density plot of the simulated C_q_ values uses a Gaussian kernel with a bandwidth of 0.01. The model density is calculated using Eq. 5 with the same parameters

The full vector of variables is ***θ*** = (*μ*, *ν*, *E*, *E*_1_, *A*, *b*_*x*_, *b*_*y*_), where *μ* is the mean number of initial molecules per partition. The overall likelihood is

5 $$ L\left(\boldsymbol{\theta}; \mathbf{c},\mathbf{x},\mathbf{y},\mathbf{n}\right)\propto p{\left(0,0;\mu, \nu \right)}^{n_0}p{\left(0,1;\mu, \nu \right)}^{n_1}\times \left\{{\displaystyle {\prod}_{j=1}^{n_2}{\displaystyle {\sum}_{\kern0.30em i=1}^{\kern0.30em m{2}^{c_0}}p\left(i,{c}_0;\mu, \nu, E,{E}_0\right)\left[\Phi \left(h,iA{G}_{c_j^{\hbox{'}}},i{A}^2{G}_{c_j^{\hbox{'}}}\left(\frac{1-E}{1+E}\right)\left({G}_{c_j^{\hbox{'}}}-1\right)\right)\right.}}\right.-\left.\left.\Phi \left(h,iA{G}_{c_j^{\hbox{'}}+\delta },i{A}^2{G}_{c_j^{\hbox{'}}+\delta}\left(\frac{1-E}{1+E}\right)\left({G}_{c_j^{\hbox{'}}+\delta }-1\right)\right)\right]\right\} $$

where *c'*_*j*_ = *c*_*j*_ − *b*_*x*_(*x* − 0.5*n*_*x*_) − *b*_*y*_(*y* − 0.5*n*_*y*_) are the detrended C_q_ data, ${G}_c={\left(1+E\right)}^{c-{c}_0}$, Φ is the distribution function of the normal distribution and *p*(*j*, *c*; *μ*, *ν*) is the probability of there being *j* molecules at cycle *c* in a partition given parameters *μ* and *ν*. The values of *p*(*j*, *c*; *μ*, *ν*) are calculated from Eq. (3) for *c* = 0 and then through repeated application of Eq. (4) for cycles up to *c*_0_. The value *c*_0_ = 6 was chosen as it is the smallest value required to achieve sufficient precision (see Fig. 2), and computational time increases rapidly as *c*_0_ increases further. See Additional file 2 for the derivation and more details.

The data comprise **n** = (*n*_0_, *n*_1_) where *n*_0_ is the count of partitions with no C_q_ value (no molecules), and *n*_1_ is the count of high censored C_q_ values (one molecule), $\mathbf{c}=\left({c}_1,\dots, {c}_{n_2}\right)$ the other C_q_ values along with $\mathbf{x}=\left({x}_1,\dots, {x}_{n_2}\right)$ and $\mathbf{y}=\left({y}_1,\dots, {y}_{n_2}\right)$ the x- and y-locations of the associated partitions. The only other data that is required is the threshold value *h*. All the data can be extracted from the dPCR experiment itself.

This model is a very good approximation as shown in Fig. 2, where a density plot of simulated data (using Eqs. (3) and (4)) almost entirely obscures the associated density plot (Eq. (5)) of the model with the same parameters.

As a Bayesian approach is being used, prior distributions are required for the parameters. We used non-informative uniform priors for *μ* and *ν*. Where suitable prior information is available gamma distributed priors could be used instead. Prior information about the efficiency *E* can be provided by preliminary quantitative PCR (qPCR) experiments. However these estimates for the qPCR efficiency are imprecise [11] and need not be the same as dPCR efficiency. For *E* we used qPCR estimates of efficiency to select a prior of Beta(190, 10) which has a mean of 0.95 and has 95 % of its mass between 0.92 and 0.98. For *E*_1_ lower values are more plausible and so a prior of Beta(18,2) was used, with mean 0.9 and 95 % of its mass between 0.74 and 0.99. For the remaining parameters there is little prior information, and so we use the non-informative priors *π*(*A*) ∝ *A* ^− 1^, *π*(*b*_*x*_) ∝ 1 and *π*(*b*_*y*_) ∝ 1.

### Single-strand adjustment

There are various reasons why *E*_1_ could be different to *E*. For example the initial molecule may be more difficult to amplify than the replicates of the target sequence because of its extra length or because of degradation. On the other hand efficiency may decrease as PCR reagents become degraded or are consumed.

Another possible factor is the presence of single-stranded DNA. In the first amplification cycle it can only be amplified to double-stranded DNA molecules, which is equivalent to double-stranded DNA failing to amplify. The standard method counts single-stranded DNA as full molecules and so if they are present it will tend to overestimate *μ* [12]. If the difference between *E* and *E*_1_ is entirely because of this issue, then the estimated parameters *Ê* and *Ê*_1_ can be used to estimate the proportion of single-stranded DNA. This leads to an estimate for *μ* given by multiplying its original estimate $\widehat{\mu}$ by an adjustment factor that is between 0.5 and 1:

6 $$ {\widehat{\mu}}_{\mathrm{adj}}=\frac{\widehat{\mu}\ }{2}\left(1+ \min \left(1,\frac{{\widehat{E}}_1}{\widehat{E}}\right)\right) $$

### Data

Full experimental details are given in [13]. Data were generated in an experiment performed by LGC on a BioMark 48.770 machine made by Fluidigm Corporation. The raw data produced by this experiment comprised fluorescence measurements made at the end of each of the 40 cycles for each partition on several chips. The chips contained 48 panels, each with 770 partitions arranged in 70 rows and 11 columns. The raw data were converted into C_q_ values for positive partitions by the ‘Fluidigm Digital PCR analysis’ software using an algorithm that is not publicly available. TheC_q_ data are provided in Additional file 3.

The experiment was performed using 3 types of DNA: Attenuated genomic DNA (gDNA), Virulent gDNA and linearised plasmid DNA. The attenuated type was *M. Tuberculosis* (MTb) H37Ra gDNA, while the virulent type was MTb H37Rv gDNA. These were both sourced from ATCC and have lengths 4,419,977 bp and 4,411,532 bp respectively. The plasmid DNA comprised a genetic construct containing the full sequences of the 16S rRNA and rpoB genes of MTb H37Rv synthesised and inserted into a pUC19 plasmid vector. It had length 8486 bp. We shall refer to these types as A, V and P respectively. Assays Jiang_16S and UCL_16S were used for the amplification of the 16S gene and their primers are described in [14] and [15], while assays GN_rpoB1 and GN_rpoB2 were used for the amplification of the rpoB gene and their primers were designed using Primer Express (Applied Biosystems). Both targets were present once in the genomes of each of the different DNA types.

There were 4 mastermixes, but only Gene Expression Mastermix (Life Technologies) was used for the present analysis. There were three dilutions (identified as 2A, 2B and 3). True values for their concentrations were not available. There were three replications of each combination of dilution, DNA type and assay, with each DNA type tested on a different chip. The fluorescent marker used was FAM and the passive reference ROX was used to normalise the measurements. ‘No template control’ panels were included and showed no issues. See [13] for more information, including the MIQE checklist [16].

### Analysis

Numerical methods are required in order to perform analysis using the model we have described. We have produced the software package edpcr for the software platform R, which was used to perform the analyses and create the plots in this paper. R can be freely downloaded from [17] and the package can be installed from within R using the command install.packages(“edpcr",repos = “http://R-Forge.R-project.org”).

The first stage of analysis is to calculate the mode of the posterior distribution via an optimisation algorithm. If a frequentist analysis is being performed rather than a Bayesian one, then no prior distributions are used and the mode is the MLE estimate for the parameters. For different initial values of *E* and *E*_1_ the optimisation algorithm may find different local maxima. We used the combinations {*E*, *E*_1_} = {0.9, 0.9}, {0.9, 0.75}, {0.85, 0.9}, {0.85, 0.75}, {0.9, 0.6} and {0.8, 0.9}, with the mode having the highest value selected as the overall mode.

A sample from the posterior density may then be produced by the random walk Metropolis algorithm [18]. The Geweke diagnostic [19] can be used to help confirm convergence.

For more information on the method of analysis see Additional file 4.

## Results and discussion

**Fig. 3:**
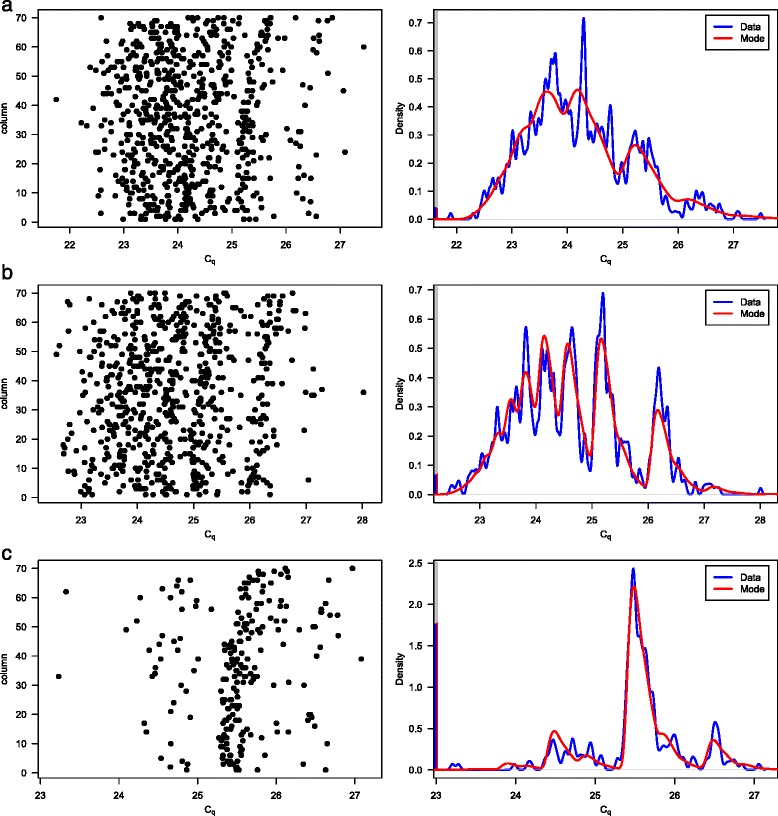
Plots of C_q_ data (left) and density plots of C_q_ data with fitted model (right). Model fit (red) shows posterior mode parameters. Data are for dilution 2A and type A (**a**), dilution 2B and type V (**b**) and dilution 3 and type P (**c**). Assay is Jiang_16S. Density plots (blue) are for detrended data (defined immediately after Eq. (4)) and use Gaussian kernels with bandwidth = 0.01. They include vertical lines representing proportion of negative partitions

Figure 3 contains plots of the C_q_ data and density plots of the detrended C_q_ data for 3 data sets. Each density plot is overlaid by the density function of the model using the posterior mode parameter estimates. The data sets are for the different dilutions and molecule types, but are each for the Jiang_16Ss assay. The model fits well to the data sets, though less well at the highest concentration, dilution 2A.

**Fig. 4:**
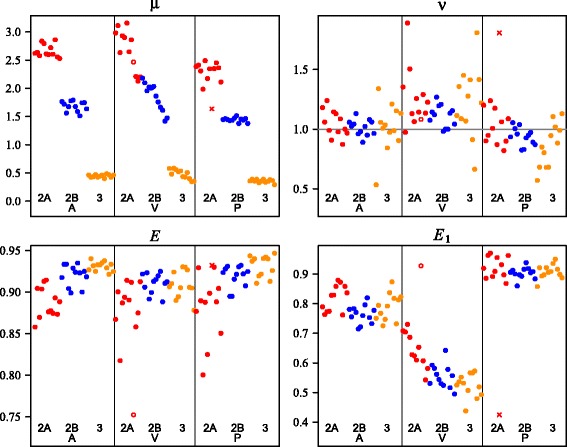
Posterior modes for *μ*, *ν*, *E* and *E*_0_. Ordered first by type (A, V, P), then dilution (2A, 2B, 3), then assay (Jiang_16S, UCL_16S, GN_rpoB1 and GN_rpoB2), then replicate (1–3). The line at *ν* = 1 represents the value *ν* takes for the Poisson distribution. The unfilled circles represent the outlier with a low mode for *E* and the cross represents the outlier with a high mode for *E*_1_

Figure 4 provides the posterior mode estimates of the parameters *μ*, *ν*, *E* and *E*_1_. The estimates are generally similar for the same type and dilution; however there are outliers, which are clearest for the *E* and *E*_1_ estimates.

If *E*_1_ is close to 1 then the peak for 1 molecule not amplified in the first cycle is small, in which case there is a risk that the parameter estimates will misalign the peaks. This appears to be the situation for the point plotted as a cross, from the type P, dilution 2A data which has a very low *E*_1_ estimate, while the other estimates from within the same group are close to 1. In that case a local mode for different starting values of *E* and *E*_1_ was consistent with the estimates for the other type P, dilution 2A data sets.

There is another outlier plotted as an unfilled circle for which the estimate of *E* is very low and the estimate of *E*_1_ is high. This appears to be due to some low outliers in the data causing a misfitting of the model, as when the mode was rerun with them censored (treated as a count of more than one molecule) the estimates were consistent with those of the other data sets.

Other possible causes of misfitting are the presence of trends that are not taken into account by the simple linear trends of the model and changes in variability. These features are typically present in the data sets to varying degrees (see changes in gradient and variability in Fig. 3), but misfitting is avoided through reasonably informative priors for *E* and *E*_1_.

### Deviation from poisson distribution

The estimates of *ν* shown in Fig. 4 provide insight about deviation from the Poisson distribution. They appear to depend on the DNA type, but not on other variables such as dilution. The medians of the estimates for the different DNA types, which are insensitive to the outliers, are 1.02 for A, 1.14 for V and 0.95 for P. Excluding the 2 outliers, the differences in the means from 1 are strongly significant for V and P with *t*-test p-values 10^−4^and 0.02 respectively, but not for A where the *t*-test *p*-value is 0.45.

**Fig. 5:**
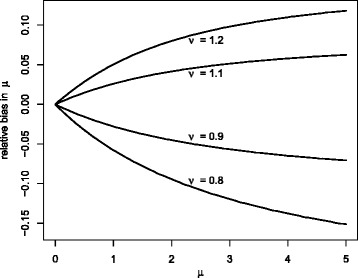
Relative bias in *μ* calculated assuming Poisson distribution for different values of *ν*. Plot is of (*μ* − [−log(*P*(*X* = 0; *μ*, *ν*))])/*μ*

**Fig. 6:**
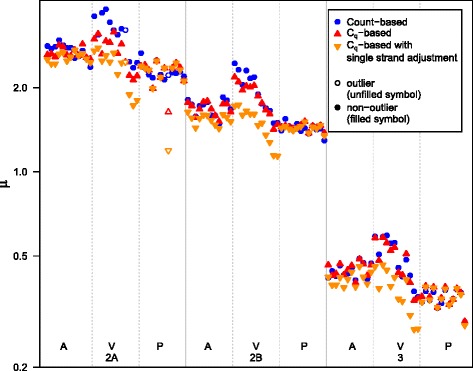
Estimates of *μ* ordered by dilution and then type. Count-based estimates use Eq. (1), C_q_-based estimates are the posterior modes for Eq. (5), while the C_q_-based estimates with single-strand adjustment are adjusted based on Eq. (6). Outliers correspond to the outliers in Fig. 4

Figure 5 illustrates the theoretical relative bias that would exist for an estimate of *μ* using the standard method due to *ν* actually taking the value 0.8, 0.9, 1.1 or 1.2. It is a plot of (*μ* − [−log(*P*(*X* = 0; *μ*, *ν*))])/*μ* where − log(*P*(*X* = 0; *μ*, *ν*)) is the estimate of the mean based on the Poisson distribution when the distribution is actually CMP with mean *μ* and dispersion *ν*. For example, if the true concentration is *μ* = 2.0 and *ν* = 1.2 then *P*(*X* = 0; *μ*, *ν*) = 0.162 so that the count-based estimate of *μ* is − log(0.162) = 1.82 and the relative bias is 0.09. The differences between the count-based and C_q_-based estimates of the concentration in Fig. 6 are consistent with these results with respect to size and sign. The issue of outliers due to misfitting the model of C_q_ data (see earlier discussion) does not affect the count-based estimates.

Figure 5 indicates that the theoretical bias due to deviation from the Poisson distribution ranges up to about 5 % over the range of concentrations examined. We have not examined concentrations above about 2.5 molecules/partition, but if similar deviation from the Poisson distribution exists for higher concentrations then based on the theoretical analysis the bias should increase. We cannot rule out greater deviation from the Poisson distribution with more substantial biases for other experiments and DNA types. In particular, It is not possible to predict how big the bias will be for droplet digital PCR (ddPCR) as the different method of partitioning the sample could lead to different values of *ν*. For ddPCR the C_q_ approach is impractical for estimating *ν*, but an indirect method for detecting the bias could be used by examining the difference in the estimate of *μ* for a range of dilutions. The retroflex method in [8] is not based on count data, and the effect of deviation from the Poisson distribution is likely to be more limited.

### Efficiencies

Figure 4 shows that the estimates for *E* are very consistent, while the estimates for *E*_1_ appear to depend on type and dilution. The biggest effect is from type. For P the estimates of *E*_1_ are close to the respective estimates for *E*, while the estimates of *E*_1_ for type A are lower, and the estimates for type V are lower still. It makes sense that the *E*_1_ estimates are higher for the plasmid DNA type as it is much shorter than the others. It is not possible to determine from the data alone how much of the differences between *E* and *E*_1_ are due to the single-strand issue. As an illustration of the effect on the estimates if the full difference are due to the single-strand issue, the adjusted estimates of *μ* (using Eq. (6)) are presented in Fig. 6 along with the count-based and C_q_-based estimates.

### MCMC results

**Fig. 7:**
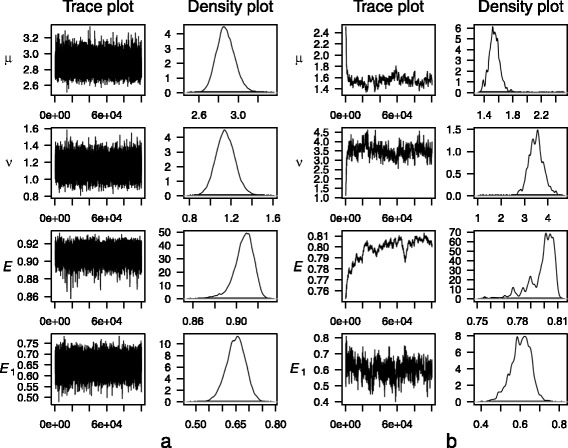
Trace and density plots for MCMC samples of posterior distributions. Data are for two of the replicate experiments for dilution 2A, type V, assay GN_rpoB1. **a** Plots for replicate 2. **b** Plots for replicate 3, for which the parameter estimates in Fig. 4 are the outliers represented by the unfilled circles in Figs. 4 and 6

MCMC samples can provide information about the posterior distribution beyond the mode, such as estimates of the mean, variance and quantiles. They can also indicate when there is a poor fit of the data, such as for the outlier with the low estimate of *E* (the unfilled circle in Fig. 4). Figure 7 contains trace and density plots for *μ*, *ν*, *E* and *E*_1_ from MCMC samples for that outlier data set and one of the other two replicates. The trace plots for the outlier move significantly from the initial values, which shows that the optimisation algorithm failed to find the mode and suggests the possibility of poor data leading to poor estimates of the mode. Examination of the data shows low outliers, and if these are censored as discussed earlier then the problems are resolved.

### Simulation

Data were simulated using the rcq function from the edpcr package. This uses Eqs. (3) and (4), with C_q_ values calculated based on exponential growth between the cycles immediately before and after the threshold was crossed. 100 data sets were simulated for each combination of *μ* = 0.5, 1.5, 2.5 or 3.5, *E*_1_ = 0.9 or 0.75 and *ν* = 0.8, 1, or 1.2. Each data set was for 770 partitions. Posterior modes for uniform priors (equivalent to MLEs) were found using Eq. (5), but excluding the linear trend parameters *b*_*x*_ and *b*_*y*_.

Results are presented in Table 1. For *μ* = 0.5 and 1.5 the C_q_-based estimates consistently have lower bias (the means are closer to the true values) and have lower standard deviation than the count-based estimates, except for *μ* = 1.5 and *ν* = 1.2 where the standard deviation is higher. The bias and standard deviation are generally better for *μ* = 2.5 and generally worse for *μ* = 3.5. This indicates good performance for concentrations up to about 2.5 copies/partition.

**Table 1:** Sample means and standard deviations of *μ*, *ν* and adjustment factor estimates for simulated data

*μ* (molecules/partition)					*ν*			.5(1 + min(1,*E*_1_/*E*))		
True value	Count-based mean	C_q_-based mean	Count-based s. d.	C_q_-based s. d.	True value	C_q_-based mean	C_q_-based s. d.	True value	C_q_-based mean	C_q_-based s. d.
0.5	0.487	**0.503**	0.026	**0.023**	0.8	0.864	0.171	0.974	0.972	0.010
1.5	1.390	**1.501**	0.066	**0.044**	0.8	0.799	0.078	0.974	0.974	0.008
2.5	2.265	**2.488**	0.110	**0.078**	0.8	0.823	0.075	0.974	0.995	0.009
3.5	3.118	**3.133**	0.174	0.308	0.8	1.080	0.163	0.974	0.980	0.070
0.5	0.487	**0.502**	0.027	**0.027**	0.8	0.864	0.195	0.895	0.893	0.014
1.5	1.388	**1.499**	0.064	**0.048**	0.8	0.806	0.092	0.895	0.895	0.011
2.5	2.270	**2.505**	0.107	**0.081**	0.8	0.804	0.083	0.895	0.915	0.020
3.5	3.107	3.053	0.168	0.337	0.8	1.094	0.176	0.895	0.987	0.028
0.5	0.498	**0.500**	0.027	**0.025**	1	1.033	0.174	0.974	0.972	0.009
1.5	1.508	**1.500**	0.064	**0.040**	1	1.022	0.090	0.974	0.974	0.006
2.5	2.511	**2.490**	0.122	**0.062**	1	1.016	0.082	0.974	0.990	0.011
3.5	3.534	3.281	0.232	**0.210**	1	1.172	0.110	0.974	0.988	0.050
0.5	0.502	**0.500**	0.031	**0.028**	1	1.059	0.195	0.895	0.894	0.015
1.5	1.498	**1.499**	0.069	**0.048**	1	1.012	0.090	0.895	0.894	0.013
2.5	2.511	2.541	0.120	0.159	1	0.979	0.102	0.895	0.899	0.030
3.5	3.565	3.180	0.237	0.314	1	1.169	0.125	0.895	0.976	0.038
0.5	0.515	**0.501**	0.030	**0.025**	1.2	1.222	0.183	0.974	0.973	0.009
1.5	1.613	**1.503**	0.075	**0.043**	1.2	1.215	0.088	0.974	0.974	0.008
2.5	2.773	**2.496**	0.152	**0.059**	1.2	1.222	0.080	0.974	0.983	0.012
3.5	3.911	**3.153**	0.243	0.579	1.2	1.296	0.114	0.974	0.978	0.042
0.5	0.510	**0.499**	0.031	**0.026**	1.2	1.208	0.167	0.895	0.895	0.013
1.5	1.613	**1.523**	0.086	0.171	1.2	1.192	0.137	0.895	0.895	0.019
2.5	2.731	**2.522**	0.125	0.173	1.2	1.174	0.113	0.895	0.896	0.030
3.5	3.961	**3.210**	0.272	0.449	1.2	1.238	0.135	0.895	0.951	0.050

Sample means and standard deviations of the count-based estimates of *μ*, using Eq. (1) and the C_q_-based estimates of *μ*, *ν* and the adjustment factor .5 (1 + min(1,*E*_1_/*E*)) from Eq. (6). Each row relates to the estimates for 100 sets of data simulated with *E* = 0.95 and a different combination of *μ*, *ν* and *E*_1_. *E*_1_ is either 0.95 (.5(1 + min(1,*E*_1_/*E*)) = 0.974) or 0.75 (.5(1 + min(1,*E*_1_/*E*)) = 0.895). Boldface is used for the C_q_-based means when they are closer to the true values than the count-based means and for the C_q_-based s. d.’s when they are smaller than the count-based s. d.’s

## Conclusions

The standard method of dPCR analysis only uses count data. In this paper we have introduced a new method of analysis that also uses the C_q_ data often produced by dPCR experiments. This method estimates the concentration of the target without the standard assumption that the initial distribution of the target is Poisson. It also produces estimates of *E*_1_ the 1st cycle amplification efficiency and *E* the subsequent amplification efficiency. Low estimates of *E* may be useful for identifying problems with the reagents. If the estimate of *E*_1_ is less than that of *E* then this may be an indication of the sample being a mixture of single-stranded and double-stranded DNA. The estimates can be used to take this into account via Eq. (6).

Our C_q_-based method was validated by simulation and demonstrated by applying it to data from different types and dilutions of DNA. Deviation from the Poisson distribution was identified for virulent and plasmid gDNA. We believe that this the first time that the Poisson distribution assumption has been tested. The bias from assuming the Poisson distribution was small for this particular case and on that basis the count-based method is still appropriate for routine applications, and the potential bias could reasonably be ignored. We do recommend caution with respect to estimates involving high concentrations, as the theoretical calculations suggest the bias could be higher (see Fig. 5). Where highly accurate quantitation is required, if the count-based method is used then an uncertainty contribution for the bias should be considered for any overall uncertainty. Further use of the C_q_-based method and other research is required to better establish the size of the biases across different types of sample and experiment, and to determine when the C_q_-based method may be prefereable. If the C_q_-based method is used, then it should only be used for concentrations up to about 2.5 molecules/partition. Application of the method could also be used as a diagnostic to identify whether *ν* is close to 1, and whether *E*_1_ is close to *E*.

## Additional files

Additional file 1: Description and illustration of normalisation method. (PDF 335 kb)
Additional file 2: Derivation of Eq. (5). (PDF 405 kb)
Additional file 3: Collated C_q_ data GE. The data used in this paper, collated from dPCR output data files. (CSV 7402 kb)
Additional file 4: Algorithm used for computational analysis. (PDF 393 kb)

## Abbreviations

CMP: Conway-Maxwell Poisson
C_q_: Quantification cycle
DNA: Deoxyribonucleic acid
dPCR: digital PCR
gDNA: genomic DNA
MCMC: Markov chain Monte Carlo
MLE: Maximum likelihood estimate
PCR: Polymerase chain reaction
qPCR: quantitative PCR
RNA: Ribonucleic acid
