# Supplementary material for: Extending digital PCR analysis by modelling quantification cycle data
Source: BMC Bioinformatics. 2016 Oct 12;17:421. doi: 10.1186/s12859-016-1275-3 (PMC5062887; doi:10.1186/s12859-016-1275-3)
Supplement: Additional file 1: — Description and illustration of normalisation method. (PDF 335 kb) [file 12859_2016_1275_MOESM1_ESM.pdf]

Description and illustration of normalisation method

Figure 1.1 illustrates the impact of normalising the fluorescence data before calculating C_q's. The normalisation uses fluorescence prior to the exponential growth phase and at the final cycle. The trends in the raw C_q data appear to be removed in the normalised Cq data, even when the gradient changes at around column 30 on the chip (1st and 3rd column of plot). The density plots for the normalised C_q data appear to show more defined peaks than those of the raw C_q data with simple linear trend removed. This suggests an improvement in the data from the normalisation. This is despite the fluorescence curves not quite reaching the plateau stage, which reduces the effectiveness of the normalisation process.

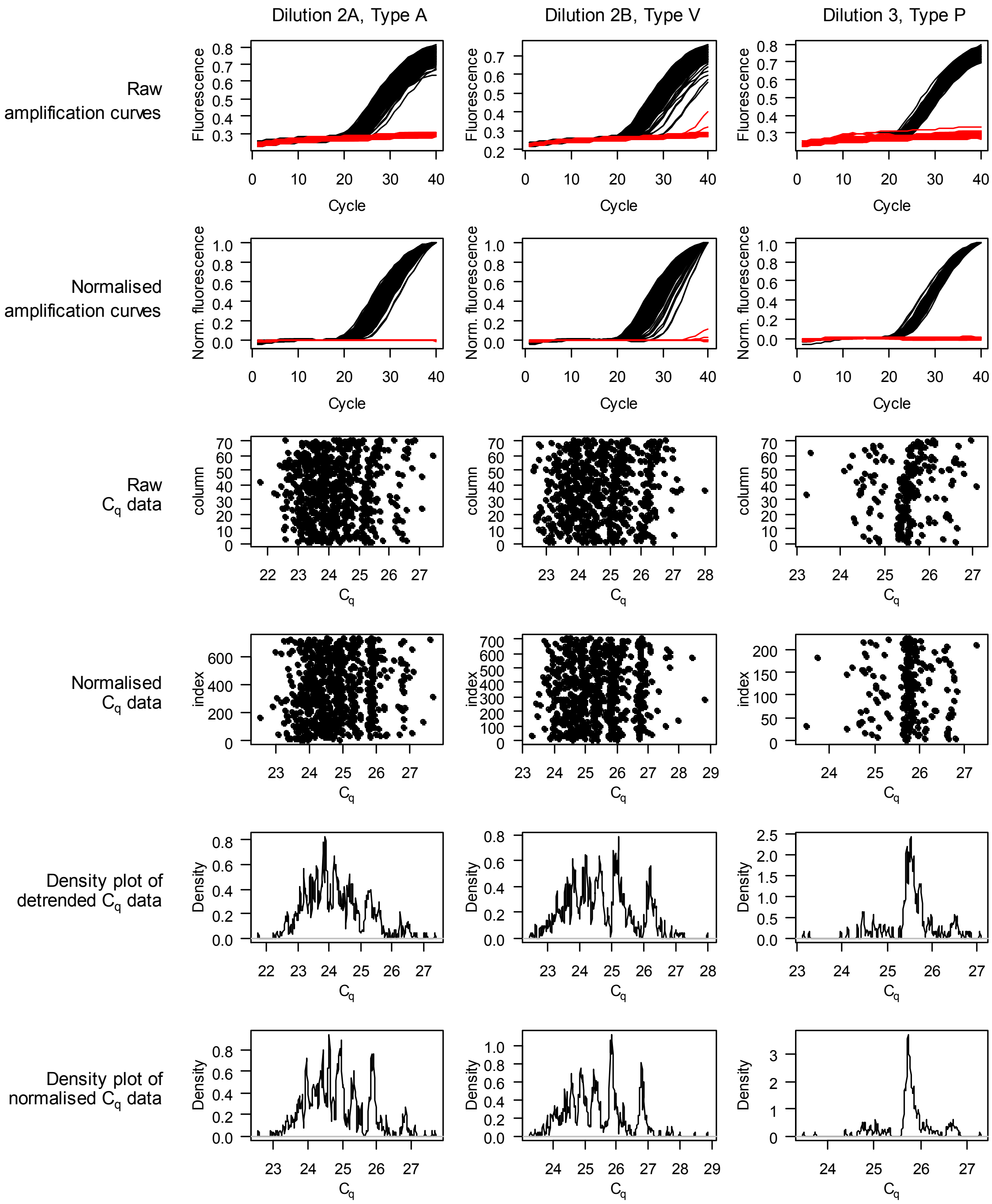

Figure 1.1. Plots comparing raw amplification curves (extracted from Fluidigm software) and normalised amplification curves with C_q data calculated from them (scatter plots and density plots) for the same 3 samples of different DNA type and concentration as in Figure 3 of main article. Density plots use Gaussian kernels with a bandwidth of 0.01.
