# Supplementary material for: Extending digital PCR analysis by modelling quantification cycle data
Source: BMC Bioinformatics. 2016 Oct 12;17:421. doi: 10.1186/s12859-016-1275-3 (PMC5062887; doi:10.1186/s12859-016-1275-3)
Supplement: Additional file 2: — Derivation of Eq. (5). (PDF 405 kb) [file 12859_2016_1275_MOESM2_ESM.pdf]

Derivation of likelihood

Initial distribution of molecules.

Let $p(j,c;\mu,\nu)$ be the probability of there being j molecules at cycle c in a partition given the parameters μ and ν. The initial distribution of parameters is given by the CMP distribution parameterised in terms of the mean μ and dispersion parameter ν, and truncated at $j = m$. Thus

$$p(j,0;\mu,\nu) = \frac{1}{Z(\lambda,\nu)}\frac{\lambda^j}{(j!)^\nu}\ j = 0,1,\dots m \text{ and } 0 \text{ otherwise, with}$$
$$\lambda = \lambda' \text{ such that } \mu = \sum_{j=0}^{m} \frac{j}{Z(\lambda',\nu)}\frac{\lambda'^j}{(j!)^\nu} \quad (1)$$

where $Z(\lambda,\nu)$ is the normalising constant and a numerical method is required to solve the second line for λ.

Let $N(c)$ be the number of molecules at cycle c. For $c > 0$

$$N(c) = N(c-1) + \mathrm{Binom}(N(c-1), E_c) \quad (2)$$

where $\mathrm{Binom}(n,p)$ represents a binomial random variable with *n* trials and probability *p* of success, and $0 < E_c < 1$. The efficiency is $E_c = E$ for $c > 1$.

Subsequent distribution of molecules.

The distribution of molecules at each cycle can then be calculated from the probabilities for the previous cycle:

$$p(j,c+1;\mu,\nu) = \sum_{i=\lceil j/2\rceil}^{j} p(i,c;\mu,\nu)\frac{(j-i)!(2i-j)!}{i!}E_j{}^{j-i}\left(1-E_j\right)^{2i-j},\ j = 1,\dots,m2^c$$

This is used, starting with the initial distribution given by Equations (1), to calculate the distribution for some cycle c_0. To extend the model to later cycles, we model the increase of the number of molecules from $N(c_0)$ as being normally distributed. We use the fact that Equation (2) represents a type of Galton-Watson branching process (Harris 2002) to derive the mean and variance:

$$\mathrm{E}(N(c)|N(c_0) = n_0) = n_0(1+E)^{c-c_0} \text{ and}$$
$$\mathrm{V}(N(c)|N(c_0) = n_0) = n_0(1-E)(1+E)^{c-c_0-1}((1+E)^{c-c_0} - 1)$$

Let $q(k;c,\boldsymbol{\theta})$ be the density of $N(c)$ where $\boldsymbol{\theta}$ represents the relevant parameters. For clarity of display we shall drop $\boldsymbol{\theta}$ until later. It can thus be approximated by

$$q(k;c) \approx \sum_{i=1}^{m2^{c_0}} p(i,c_0;\mu,\nu)\phi\big(k, i(1+E)^{c-c_0}, i(1-E)(1+E)^{c-c_0-1}((1+E)^{c-c_0}-1)\big) \quad (3)$$

where $\phi(x,a,\sigma^2) = \frac{1}{\sqrt{2\pi\sigma^2}}e^{-\frac{(x-a)^2}{2\sigma^2}}$ is the density of the normal distribution.

Model of fluorescence

Let $R(c)$ be the relative fluorescence at cycle c with density $g(x;c)$. We model the relationship between $R(c)$ and $N(c)$ by

$$R(c) = A \times N(c),$$

where $A > 0$ is the relative fluorescence per molecule. Then

$$g(x;c) \approx$$

$$\sum_{i=1}^{m2^{c_0}} p(i,c_0;\mu,\nu,E)\phi\big(x, Ai(1+E)^{c-c_0}, A^2 i(1-E)(1+E)^{c-c_0-1}((1+E)^{c-c_0}-1)\big) \quad (4)$$

Basic model of threshold cycle

Let $C(h)$ be the quantification cycle for threshold h with density $f(c;h)$. The fluorescence reaching the threshold h earlier than c is equivalent to the fluorescence being more than h at cycle c. Thus the probabilities of these events are equal so that

$$f(c;h) = \frac{d}{dc}P(C(h) < c) = \frac{d}{dc}P(R(c) > h)$$

A finite difference approximation can be used for the differentiation to approximate $f(c;h)$ in terms of $g(x;c)$.

$$\begin{aligned} f(c;h) &= \frac{d}{dc}P(X(c) > h) \approx \delta^{-1}[P(X(c) < h) - P(X(c+\delta) < h)] \\ &\approx \int_{-\infty}^{h} g(x;c)dx - \int_{-\infty}^{h} g(x;c+\delta)dx \end{aligned} \quad (5)$$

where $\delta > 0$ is a small positive constant.

Detrending

Let b_x and b_y be parameters representing the trends in the x- and y-directions respectively. The detrended data are then given by

$$\tilde{c_j} = c_j - b_x\left(x - \frac{n_x}{2}\right) - b_y\left(y - \frac{n_y}{2}\right) \quad (6)$$

where x is the column number out of n_x and y the row number out of n_y.

Combining Equations (4), (5) and (6) $f(c;h)$ can be approximated by

$$f(\mathrm{c};h) \approx \sum_{i=1}^{m2^{c_0}} p(i,c_0;\mu,\nu,E,E_0)\left[\Phi\left(h,iAG_{\tilde{c}},iA^2G_{\tilde{c_j}}\left(\frac{1-E}{1+E}\right)(G_{\tilde{c}}-1)\right)-\Phi\left(h,iAG_{\tilde{c}+\delta},iA^2G_{\tilde{c}+\delta}\left(\frac{1-E}{1+E}\right)(G_{\tilde{c}+\delta}-1)\right)\right] \quad (7)$$

where $G_c = (1+E)^{c-c_0}$.

Censoring

Let $\mathbf{n} = (n_0, n_1)$ where n_0 is the number of negative partitions (no molecules) and n_1 is the count of high censored C_q values (one molecule). Let $\mathbf{c} = (c_1, \ldots, c_{n_2})$ be the uncensored C_q values along with $\mathbf{x} = (x_1, \ldots, x_{n_2})$ and $\mathbf{y} = (y_1, \ldots, y_{n_2})$ the x- and y-locations of the associated partitions. The total number of partitions is $n = n_0 + n_1 + n_2$.

Overall likelihood

The overall likelihood is the product of the densities of Equation (7) for the C_q data and the probabilities from Equations (1) of the count data for the remaining partitions,

$$L(\boldsymbol{\theta};\mathbf{c},\mathbf{x},\mathbf{y},\mathbf{n}) \propto p(0{,}0;\mu,\nu)^{n_0} p(0{,}1;\mu,\nu)^{n_1} \times$$

$$\left\{\prod_{j=1}^{n_2}\sum_{i=1}^{m2^{c_0}} p(i,c_0;\mu,\nu,E,E_0)\left[\Phi\left(h,iAG_{\tilde{c_j}},iA^2G_{\tilde{c_j}}\left(\frac{1-E}{1+E}\right)\left(G_{\tilde{c_j}}-1\right)\right)-\right.\right.$$
$$\left.\left.\Phi\left(h,iAG_{\tilde{c_j}+\delta},iA^2G_{\tilde{c_j}+\delta}\left(\frac{1-E}{1+E}\right)\left(G_{\tilde{c_j}+\delta}-1\right)\right)\right]\right\} \quad (8)$$

where $\Phi(x,\mu,\sigma^2) = \int_{-\infty}^{x}\phi(s,\mu,\sigma^2)ds$ is the distribution function of the normal distribution.
