# Supplementary material for: Extending digital PCR analysis by modelling quantification cycle data
Source: BMC Bioinformatics. 2016 Oct 12;17:421. doi: 10.1186/s12859-016-1275-3 (PMC5062887; doi:10.1186/s12859-016-1275-3)
Supplement: Additional file 4: — Algorithm used for computational analysis. (PDF 393 kb) [file 12859_2016_1275_MOESM4_ESM.pdf]

Algorithm

The posterior distribution for are $\boldsymbol{\theta} = \left(\mu, \nu, E, E_0, A, b_x, b_y\right)$ given the data is

$$\pi(\boldsymbol{\theta}|\mathbf{c}, \mathbf{x}, \mathbf{y}, \mathbf{n}) \propto L(\boldsymbol{\theta}; \mathbf{c}, \mathbf{x}, \mathbf{y}, \mathbf{n})\pi(\boldsymbol{\theta})$$

with the likelihood

$$L(\boldsymbol{\theta}; \mathbf{c}, \mathbf{x}, \mathbf{y}, \mathbf{n}) \propto p(0,0; \mu, \nu)^{n_0} p(0,1; \mu, \nu)^{n_1} \times$$
$$\left\{\prod_{j=1}^{n_2} \sum_{i=1}^{m2^{c_0}} p(i, c_0; \mu, \nu, E, E_0) \left[\Phi\left(h, iAG_{\tilde{c_j}}, iA^2 G_{\tilde{c_j}}\left(\frac{1-E}{1+E}\right)\left(G_{\tilde{c_j}} - 1\right)\right) - \right.\right.$$
$$\left.\left.\Phi\left(h, iAG_{\tilde{c_j}+\delta}, iA^2 G_{\tilde{c_j}+\delta}\left(\frac{1-E}{1+E}\right)\left(G_{\tilde{c_j}+\delta} - 1\right)\right)\right]\right\}$$

where $\Phi(x, \mu, \sigma^2) = \int_{-\infty}^{x} \phi(s, \mu, \sigma^2) ds$ is the distribution function of the normal distribution. See 'Additional file 1.pdf' for derivation and more details.

The joint prior distribution $\pi(\boldsymbol{\theta})$ is the product of the individual prior distributions given by

$$\mu \sim \Gamma(\alpha_1, \beta_1)$$
$$\nu \sim \Gamma(\alpha_2, \beta_2)$$
$$E \sim \text{Beta}(\alpha_3, \beta_3)$$
$$E_1 \sim \text{Beta}(\alpha_4, \beta_4)$$
$$\pi(b_x) \propto 1$$
$$\pi\left(b_y\right) \propto 1$$
$$\pi(A) \propto A^{-1}$$

for values of $\alpha_1, \dots, \alpha_4, \beta_1, \dots, \beta_4$ that reflect prior opinion regarding the associated parameters. See main paper for more details.

The function used to transform $\boldsymbol{\theta}$ to $\boldsymbol{\varphi} = \left(\mu', \nu', E', {E_1}', A', b_x, b_y\right)$ is:

$$\boldsymbol{\varphi} = f(\boldsymbol{\theta}) = \left(\log(\mu), \log(\nu), -\log\left(\frac{1}{E} - 1\right), -\log\left(\frac{1}{E_0} - 1\right), \frac{\log(A)}{\log(1+E)}, b_x, b_y\right), \quad (1)$$

with the inverse given by

$$\boldsymbol{\theta} = f^{-1}(\boldsymbol{\varphi}) = \left(e^{\mu'}, e^{\nu'}, \left(1 + e^{-E'}\right)^{-1}, \left(1 + e^{-{E_1}'}\right)^{-1}, (1 + E')^{A'}, b_x, b_y\right), \quad (2)$$

The posterior in terms of the transformed parameters is given by

$$\pi(\boldsymbol{\varphi}|\mathbf{c}, \mathbf{x}, \mathbf{y}, \mathbf{n}) = \pi(f^{-1}(\boldsymbol{\theta})|\mathbf{c}, \mathbf{x}, \mathbf{y}, \mathbf{n}) \times \mu\nu(1 + E)E_1(1 - E_1)\log(1 + E)A$$

The algorithm for producing the MCMC chain is as follows in which N is the length of the chain:

1. For each combination of $\tilde{E}, \tilde{E}_1$ calculate the initial parameter estimates

$$\widetilde{\boldsymbol{\theta}} = \left(\tilde{\mu} = -\log\left(\frac{n_0}{n}\right), \tilde{\nu} = 1, \tilde{E}, \tilde{E}_1, \tilde{A} = he^{\frac{0.6n_0}{n_0+n}}\left(1+\tilde{E}_1\right)^{-1}\left(1+\tilde{E}\right)^{1-\bar{c}}, \tilde{b}_x, \tilde{b}_y\right)$$

   (a) Transform from $\widetilde{\boldsymbol{\theta}}$ to $\widetilde{\boldsymbol{\varphi}}$ using Equation (1).

   (b) Estimate the local maximum $\boldsymbol{\varphi}_{\text{mode}}$ using the Nelder-Mead algorithm starting at $\widetilde{\boldsymbol{\varphi}}$

2. From the results of step 1 identify the overall mode $\boldsymbol{\varphi}_{\text{mode}}$;
3. Estimate the Hessian matrix $\mathbf{H}$ at $\boldsymbol{\varphi}_{\text{mode}}$, and use the matrix to estimate the covariance matrix $\boldsymbol{\Sigma}_{\text{mode}} = \mathbf{H}^{-1}$. (If **H** is not invertible reduce the elements on its diagonal so that it is.)
4. Set $\boldsymbol{\varphi}^{(1)} = \boldsymbol{\varphi}_{\text{mode}}$;
5. For $i = 2 : N$
   (a) Sample u from Uniform$(0,1)$
   (b) Sample $\boldsymbol{\varphi}^*$ from $N\left(\boldsymbol{\varphi}^{(i-1)}, \boldsymbol{\Sigma}_{\text{mode}}\right)$
   (c) If

$$u < \min\left\{1, \frac{\pi(\boldsymbol{\varphi}^*|\mathbf{c},\mathbf{x},\mathbf{y},\mathbf{n})}{\pi(\boldsymbol{\varphi}^{(i-1)}|\mathbf{c},\mathbf{x},\mathbf{y},\mathbf{n})}\right\}$$

   then

$$\boldsymbol{\varphi}^{(i)} = \boldsymbol{\varphi}^*$$

   otherwise

$$\boldsymbol{\varphi}^{(i)} = \boldsymbol{\varphi}^{(i-1)}$$

6. Transform values $\boldsymbol{\varphi}^{(1)}, \ldots, \boldsymbol{\varphi}^{(N)}$ back to $\boldsymbol{\theta}^{(1)}, \ldots, \boldsymbol{\theta}^{(N)}$ using Equations (2).

Steps 3-5 are performed by the R function MCMCmetrop1R from the MCMCpack package.
